# Resurrection ecology in *Artemia*

**DOI:** 10.1111/eva.12522

**Published:** 2017-10-23

**Authors:** Thomas Lenormand, Odrade Nougué, Roula Jabbour‐Zahab, Fabien Arnaud, Laurent Dezileau, Luis‐Miguel Chevin, Marta I. Sánchez

**Affiliations:** ^1^ CEFE UMR 5175 CNRS, Université de Montpellier, Université Paul‐Valéry Montpellier Montpellier Cedex 5 France; ^2^ Laboratoire EDYTEM UMR 5204 du CNRS, Environnements, Dynamiques et Territoires de la Montagne, Université de Savoie Le Bourget du Lac Cedex France; ^3^ Géosciences Montpellier, UMR 5243 Université de Montpellier Montpellier Cedex 05 France; ^4^ Estación Biológica de Doñana (CSIC) Sevilla Spain

**Keywords:** biological invasions, cysts, global change, long‐term adaptation, sediment core

## Abstract

Resurrection ecology (RE) is a very powerful approach to address a wide range of question in ecology and evolution. This approach rests on using appropriate model systems, and only few are known to be available. In this study, we show that *Artemia* has multiple attractive features (short generation time, cyst bank and collections, well‐documented phylogeography, and ecology) for a good RE model. We show in detail with a case study how cysts can be recovered from sediments to document the history and dynamics of a biological invasion. We finally discuss with precise examples the many RE possibilities with this model system: adaptation to climate change, to pollution, to parasites, to invaders and evolution of reproductive systems.

## 1 INTRODUCTION

Many aquatic invertebrate taxa, such as Branchiopoda, Ostracoda, or Copepoda, depend on the production of long‐lived dormant stages, which allow them to persist under unfavorable conditions (drought, high temperatures, extreme salinities, predation, or food scarcity), especially in highly fluctuating environments (Brendonck, 1996; Hairston, 1996; Hand, Denlinger, Podrabsky, & Roy, 2016; Wiggins, Mackay, & Smith, 1980). These dormant stages form egg banks that can be stored in the environment, allowing for an escape strategy in the form of dispersal through time (Venable & Lawlor, 1980). From a practical or applied point of view, because dormant stages can remain viable or at least carry genetic information for decades or even centuries in sediments (Hairston, Ellner, & Kearns, 1996) or in laboratory collections, they can provide researchers with natural archives of recent evolutionary and ecological histories of populations (Brendonck & De Meester, 2003; Kerfoot, Robbins, & Weider, 1999; Weider, Lampert, Wessels, Colbourne, & Limburg, 1997).

With the exception of a handful of long‐term field studies, contemporary evolutionary ecology strongly relies on spatial comparison among populations. These spatial studies are often used to extrapolate about temporal predictions using the so‐called space‐for‐time substitution (Etterson & Shaw, 2001; Pickett, 1989). However, an alternative way to directly capture the temporal component of evolutionary responses of populations without relying on costly long‐term monitoring is by studying ancient populations from propagule banks. This is the approach taken by the emerging field of “resurrection ecology” (Experimental Paleoecology, Kerfoot et al., 1999; Kerfoot & Weider, 2004; thereafter RE). Past populations can be studied by reviving these propagule banks (Etterson et al., 2016; Orsini et al., 2013) or by studying these banks genetically (Angeler, 2007). For example, time‐shift experiments can be used to study temporal adaptation by comparing the fitness of a population under current, past, or future environmental conditions (Blanquart & Gandon, 2013; Gaba & Ebert, 2009). Because of their dormant stages, a number of aquatic animals are ideally suited for “resurrection ecology,” and, as we will see, *Artemia* is a particularly attractive one, although very limited attempts have been made to exploit its full potential.

Resurrection ecology has been used to study many important questions in ecology and evolution: male–female coevolution (Rode, Charmantier, & Lenormand, 2011), host–parasite coevolution (Decaestecker et al., 2007; Frickel, Sieber, & Becks, 2016; Gaba & Ebert, 2009; Thrall et al., 2012), spatiotemporal variability in community and trophic structure (Anderson & Battarbee, 1994; Vandekerkhove et al., 2005), biological invasions (Kerfoot, Ma, Lorence, & Weider, 2004), and biodiversity dynamics (Gregory‐Eaves & Beisner, 2011). RE can also be particularly useful to study the dynamics of adaptation to changing environments (Bradshaw & Holzapfel, 2001; Frisch et al., 2014; Gaba & Ebert, 2009; Hairston et al., 1999; Pulido & Berthold, 2010) and hence provide better predictions of adaptive responses to global change (Hoffmann & Sgro, 2011). Most RE studies to date have been conducted on diapausing eggs of cladocerans (Angeler, 2007; Decaestecker et al., 2007; Hairston et al., 1999; Miner, De Meester, Pfrender, Lampert, & Hairston, 2012; Orsini, Spanier, & De Meester, 2012; Roy Chowdhury, 2016; Weider et al., 1997), mainly on *Daphnia*, in freshwater ecosystems. The scarcity of studies in other organisms and environments has been highlighted as an important limitation of RE (Angeler, 2007). Identifying new study systems is therefore essential to fully exploit the potential of RE to address evolutionary and ecological questions.

In this study, we present the hypersaline macrocrustacean brine shrimp *Artemia* (Crustacea, Branchiopoda, Anostraca) as a valuable model system for RE. We start by reviewing the biological and ecological characteristics that make *Artemia* an interesting model in RE. We then present how *Artemia* egg banks can be obtained from sediment cores, with an example of such an approach from our own work in the Aigues‐Mortes saltern in South France. Overall, we discuss for the first time the usefulness and limitations of this model system for RE.

### 1.1 The biology and ecology of *Artemia*

*Artemia* is among the most intensely studied aquatic organisms, due to its importance in aquaculture and its broad use as a model system in ecotoxicology, developmental biology, ecology and evolutionary biology. It is an extreme halophilic organism occurring in hypersaline environments such as salt pans, inland salt lakes, and coastal salt lagoons worldwide (Lenz & Browne, 1991). The genus includes seven sexual species and several parthenogenetic strains of different ploidy (Baxevanis, Kappas, & Abatzopoulos, 2006; Gajardo, Abatzopoulos, Kappas, & Beardmore, 2002). Parthenogenetic populations are widely distributed except in the Americas. Sexual and parthenogenetic *Artemia* tend to occupy different microhabitats and to be temporally segregated (Agh et al., 2007), although a handful of populations are mixed (Amat, Hontoria, Navarro, Vieira, & Mura, 2007). The latter are particularly interesting to study a diversity of evolutionary and ecological processes.

From an ecological point of view, *Artemia* is a keystone taxon in hypersaline food webs, where it constitutes the dominant or exclusive macrozooplankton. It is the main prey for aquatic birds (Sánchez, Green, & Castellanos, 2006), the intermediate host for many species of parasites (Georgiev et al., 2005; Rode, Landes, et al., 2013; Rode, Lievens, et al., 2013; Vasileva et al., 2009), and the main consumer of phytoplankton. Because hypersaline ecosystems are simplified, and because of its central role in these ecosystems, *Artemia* is particularly suited to study cascading effects (Sánchez, Paredes, Lebouvier, & Green, 2016). For instance, it is a good system to explore how genetic changes in one species translate into “cascading” effects throughout the community and ecosystem.

*Artemia* life history is well studied (Amat et al., 2007; Browne, Davis, & Sallee, 1988; Browne, Sallee, Grosch, Segreti, & Purser, 1984; Browne & Wanigasekera, 2000; Shirdhankar, Thomas, & Barve, 2004). It is characterized by a short generation time, reaching maturity through a series of approximately 15 molts, in less than 20 days (Jensen, 1918), and it has high fecundity, producing up to 250 embryos per brood (Amat et al., 2007). These are key attributes for a good RE model, as it makes it easy to detect evolutionary changes within short periods of time (Hairston et al., 1999; Kerfoot & Weider, 2004; Kerfoot et al., 1999). *Artemia* females produce free‐living nauplii (ovoviviparity) when environmental conditions are favorable, and produce dormant encysted embryos or diapausing cysts (oviparity) under adverse conditions (i.e., high salinity and temperature, low oxygen levels, and food supply or short photoperiods among others (Criel & Macrae, 2002; Clegg & Trotman, 2002; Nambu, Tanaka, & Nambu, 2004)).

### 1.2 The *Artemia* cyst

The cyst is a key feature of *Artemia* as an important RE model. Cyst production is high in hypersaline ecosystems, reaching hundreds or thousands of metric tons per year (e.g., in the Great Salt Lake, Clegg & Jackson, 1998). Cysts accumulate in sediments as eggs banks (Fig. S1) and can be recorded in sediment cores dated back to 200,000 years (Djamali et al., 2010). Cysts are commercially harvested for aquaculture (Sorgeloos, 1980). They can also be stored in laboratory cyst banks for research purposes (e.g., the cyst bank of the Laboratory of Aquaculture and *Artemia* Reference Center, ARC). Cysts preserved in airtight drums under cold storage conditions (4°C) can remain viable for long periods and can be hatched following well‐known standardized protocols (Sorgeloos, Persoone, Baeza‐Mesa, Bossuyt, & Bruggeman, 1978). Cysts of *Artemia* are considered among the most resistant of all animal life history stages to extreme environmental conditions (Clegg & Jackson, 1998; Clegg & Trotman, 2002; Hand & Podrabsky, 2000). They are able to tolerate high levels of UV radiation, prolonged anoxia, extreme temperatures, and repeated cycles of hydration and severe desiccation (Clegg, 2001, 2005; Clegg & Conte, 1980; Liang & MacRae, 1999; Warner, 1989). These are normal conditions that cysts encounter from the moment that they are released from the female into the water, accumulate along the shores, and become covered by sediments. Several features are responsible for such impressive stress resistance of cysts: the disaccharide trehalose, the small heat‐shock protein and molecular chaperone p26, and the RNA‐binding protein Artemin with RNA chaperone activity (Clegg, 1986; Crowe, Clegg, & Crowe, 1998; Warner, Brunet, MacRae, & Clegg, 2004). The cyst shell is also decisive in protecting the embryo from mechanical damage (Clegg, 2005; Clegg & Conte, 1980), UV radiation (Tanguay, Reyes, & Clegg, 2004), desiccation (Clegg, 2005), and resistance to microbial and hydrolytic damage, thus protecting the DNA inside the cyst (Clegg & Conte, 1980).

Entire cysts have been recovered from sediment cores as old as 27,000 years ago in the Great Salt Lake, Utah, USA (Clegg & Jackson, 1997). The extraordinary stability of encysted embryos has led researchers to consider *Artemia* as a unique model system to study ancient DNA in paleoecological research (Clegg & Jackson, 1997). For example, Manaffar et al. (2011) analyzed molecularly (by sequencing the exon 7 of the Na/K ATPase gene) the cysts bank of Lake Urmia (Iran) with radiocarbon ages estimated at ~5,000 years. This analysis showed that this ancient population was dominated by *A. parthenogenetica* (with some *A. urmiana*). Comparison with the present day niche of those species suggests that the past lake condition was brackish. Similar studies have been made using more recent cysts. Motivated by the intriguing finding of parthenogenetic individuals in a commercial sample from the Great Salt Lake, Utah, USA (Campos‐Ramos et al., 2003), a population usually described as being exclusively made of *Artemia franciscana,* Endebu et al. (2013) analyzed molecularly, cysts samples collected between 1997 and 2005. They showed that indeed, there has been a period where the two species coexisted.

Additional techniques have been proposed to analyze historical cyst samples. Nielson and Bowen (2010) studied the hydrogen and oxygen isotope ratios of the common structural biopolymer chitin in *Artemia* (present in cysts and free‐living stages). They suggested that it would be a powerful tool for paleoenvironmental and paleoecological reconstruction, showing that these measures were a good indicator of ecological and biogeochemical changes within lakes. Despite the use of *Artemia* in paleoecology and paleobiology during the last decade, little attention has so far been paid to its potential use in RE (but see Rode et al., 2011 and below). Cysts from laboratory collections can remain viable for decades (Abatzopoulos, Kappas, Bossier, Sorgeloos, & Beardmore, 2002) making it possible to revive ancient and present populations over significant temporal scales and use them in experiments. However, much less is known about cyst viability and DNA quality in cysts recovered from sediments. We present below a study addressing these questions.

### 1.3 Origin of cysts for RE studies

Several *Artemia* cyst collections have been established in different laboratories (e.g., Laboratory of Aquaculture & *Artemia* Reference Center (ARC) at Ghent University in Belgium and the Institute of Aquaculture of “Torre de la Sal” (CSIC) in Castellón, Spain). Cysts kept in good conditions in these collections have a much higher hatchability than sediment‐collected cysts. Moreover, collections contain *Artemia* populations from all over the world (the five continents, Sorgeloos, Lavens, Leger, & Tackaert, 1990), thus allowing studies at broad spatial scales, with information about biological parameters of populations and environmental conditions of the collections sites. These collections are readily available for *Artemia*, are abundant, and span several decades. In comparison, such an approach has been advocated, but only recently initiated, for example, in plants (Etterson et al., 2016). These collections are invaluable and require long‐term maintenance and constant archiving and development (Sorgeloos et al., 1990). The time horizon for cysts hatchability is not entirely clear under these conditions, but even if precise samples may not be enough to obtain complete time series in most populations, these collections offer an excellent starting point for RE studies. These collections can be also exploited from a conservation perspective using them to restore locally extirpated populations (Muñoz et al., 2008), given the dramatic decrease in *Artemia* biodiversity at a global scale (Amat et al., 2007).

Much less is known about *Artemia* cysts collected from natural sediment. Very few studies have been conducted in this direction, even though obtaining such samples could extend by far the utility of *Artemia* as a model system in RE studies. Obtaining and dating such samples can be challenging. We develop in the next section, how this can be achieved, from a case study in the Aigues‐Mortes saltern in South France.

## 2 SAMPLING AND DATING CYSTS FROM SEDIMENTS

The objective was to document the invasion of *A. franciscana* (hereafter *Af*) in the Aigues‐Mortes saltern in southern France, since its introduction in 1970 and to obtain temporal series of cysts of both the local population (*A. parthenogenetica,* hereafter *Ap*) and the invading *Af* species. In contrast to the bottom of lakes, surface sedimentation in commercial salterns can be much more erratic and heavily disturbed by human activity. Yet, these sediments form a very important fraction of *Artemia* habitats. We chose a favorable sampling site based on prior local historical information. In the saltern, water circulates from the sea to the different ponds by channels. The ponds are connected to the channels through sluice gates. The site we chose (43°31′24.72″N; 4°14′12.67″E) was close to an ancient gate that connected a pond and a channel. It was also located on a shore where cysts tended to accumulate due to the direction of dominant wind. This sluice gate was shut down in the early 1960s (D. Facca, personal communication) and silted up ever since. Cysts accumulated in the sediments, creating a time series. The site is also well connected to the water circuit in the rest of the saltern, such that cysts collected there are representative of the *Artemia* population in the entire saltern. Four cores were sampled (site map on Fig. S2). The first one (ABB12) was obtained on February 1, 2012, and was used to follow the invasion of *Af* in the saltern. The other three cores (ABB13_P5 P6 and P7) were obtained on June 26, 2013 using a manual corer and were used to finely study sedimentation patterns in the saltern.

### 2.1 Cyst quality along the first sediment core

First, the proportion of cysts that could not be molecularly identified in ABB12 core increased from 10% at the surface to 95% at the bottom of the core. This indicates that DNA quality declines with increasing time in the sediments. This does not prevent one from performing molecular analyses, as many cysts are available at each horizon in the cores. However, because of this degradation, methodologies involving long DNA fragments may be difficult to implement. For instance, in the samples taken below 40 cm, DNA fragments were generally smaller than 200 base pairs (bp), which is too small for microsatellite amplification in *Artemia* (Fig. S3), but sufficient to perform PCR on short diagnostic sequences or for direct sequencing of short fragments.

**Figure 1:**
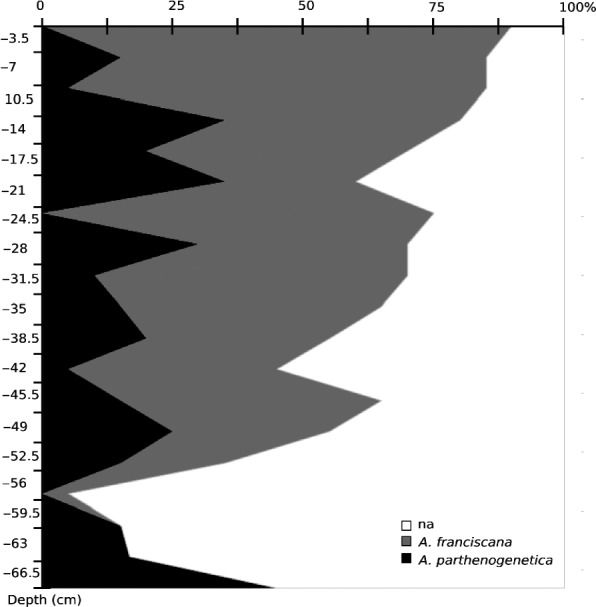
Proportion of *Artemia parthenogenetica* and *Artemia franciscana* along the core horizons. na: nonidentifiable cyst; *A. franciscana* and *A. parthenogenetica*: cyst identified as sexual and nonsexual species. Data from ABB12 core

Among cysts that could be identified molecularly, the proportion of *Af* increases through time (Figure 1), from 100% in the top horizon to 0% in the last three horizons. This variation reflects the invasion of *Af* in the saltern since its introduction. Note that although the 20 cysts collected on the surface were *Af*,* Ap* is still present in the saltern today (Nougué et al., 2015). *Af* was intentionally introduced in 1970 (D. Facca, personal communication), which indicates that most of the cores (down to 60 cm depth) were after this date. Hatching success decreased sharply below the surface layer (12.5% at surface, 9.5% and 0.3% in layers 1 and 2, and no success below).

### 2.2 Sediment dating and core synchronization

**Figure 2:**
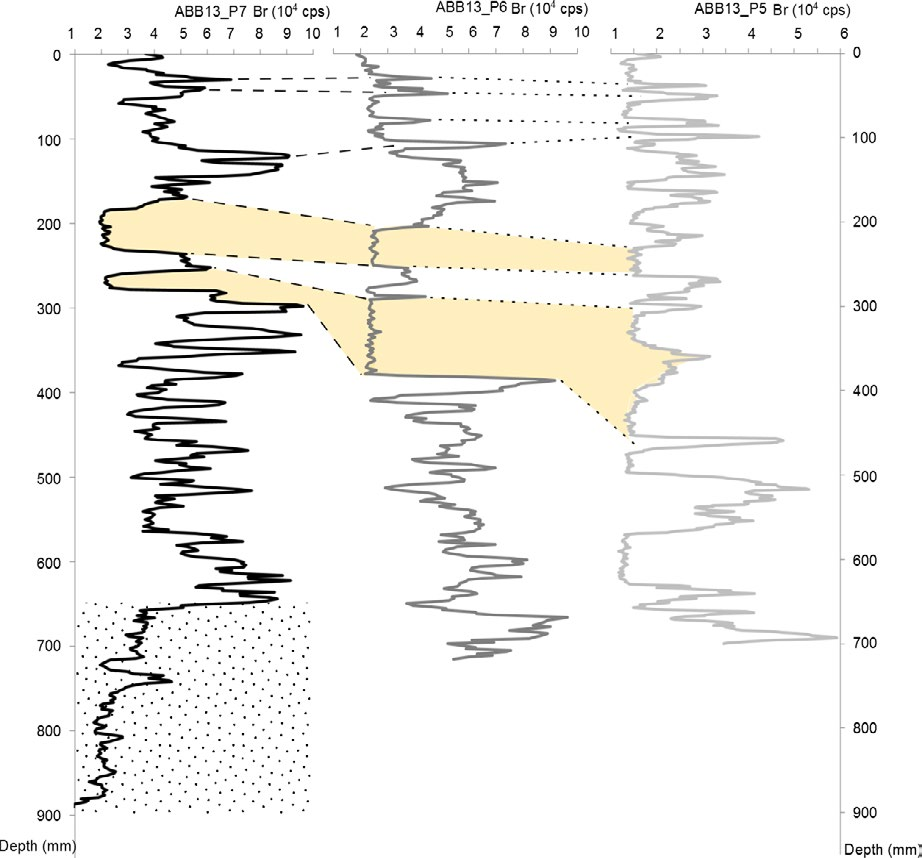
Core correlation using bromine concentration along the cores horizons. Horizontal axis corresponds to the counts per second (cps) recorded at 30 kV for bromine, while vertical axis corresponds to the depth in millimeter for each core. On ABB13_P7 core, the dotted/shaded zone corresponds to the bottom of the core where sediments were stirred. Yellow shaded areas mark the sand area spotted in the cores. Dashed lines are correlations between ABB13_P7 and ABB13_P6, while dotted lines show correspondence between ABB13_P6 and ABB13_P5

Visual observation of the cores (Fig. S5) revealed that only ABB13_P6 and ABB13_P7 contained the complete sediment sequence that followed the sluice gate closure. Indeed, in the P6 and P7 cores stirred sediments with no horizons are visible from 81 to 96 cm and 67 to 90 cm, respectively. They correspond to the bottom of the channel that was actively maintained before the gate was definitively closed. No regular patterns of sedimentation (e.g., like annual cycles) were observable in the cores. However, two sandy horizons were visible in the three cores. X‐ray fluorescence observations showed alternation in organic matter containing elements such as sulfur, copper, and bromine and sands containing silicon. The PCA (Fig. S4) confirms this major source of heterogeneity among horizons by exhibiting a first PCA axis (41% of the variance), segregating elements corresponding to organic versus nonorganic matter (clay and sandstone). The second PCA axis (28%) differentiates between elements representing low versus high oxygenated organic matter. As shown in Figure 2, the three cores present alternative horizons with high and low quantities of bromine (element present in organic matter). The two sandy horizons that were visually observable are also observable on the three bromine graphs (yellow zones in Figure 2), as well as the stirred sediments in the bottom of ABB13_P7 (dotted‐shaded zone in Figure 2).

**Figure 3:**
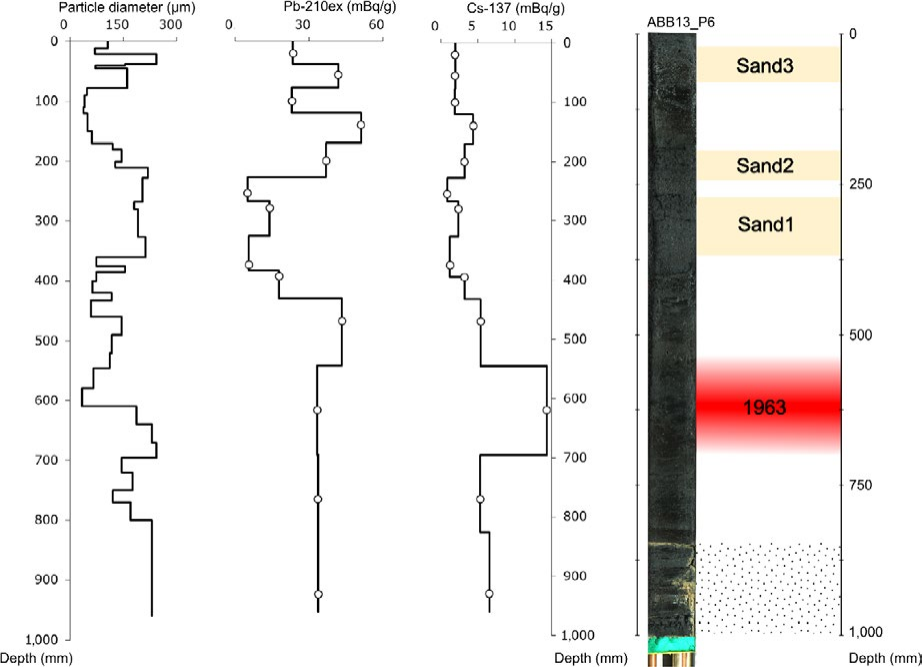
ABB13_P6 core dating. To distinguish between recent and older core horizons, we used both continuous time detection (particle diameter and ^210^Pb) and the detection of event markers (^137^Cs)

To distinguish between recent and older core horizons (Figure 3), we used both continuous time detection (particle diameter and ^210^Pb) and the detection of event markers (^137^Cs). The ^210^Pb signal usually follows a logarithmic decay in sediment. However, ^210^Pb tends to be washed away from sandy sediments, making this radionuclide difficult to interpret in our cores where different layers exhibit strong heterogeneity in sand content. Particle diameter is negatively correlated with bromine content (Figure 2). The signal for the two major sandstone horizons is visible with high‐diameter particles from 20 to 35 cm deep (labeled Sand 1 and Sand 2 in Figure 3). Another high‐diameter‐particle sandy horizon (labeled Sand 3 hereafter) was detected between 5 and 10 cm (Figure 3), matching with low bromine density in ABB13_P6 (Figure 2). ^137^Cs was detected in all layers, indicating that the entire time series was probably post‐1954. A maximum of 14.35 mBq/g was detected at 62 cm deep, signaling the fallout maximum of 1963. However, we were not able to detect the Chernobyl peak of 1986. Three possible reasons are as follows: (i) the scale of analysis was too coarse for such a singular event; (ii) the time series stops prior to 1986. In the first case, a finer timescale sampling might be realized especially between 10 and 20 cm depth, where the quantity of ^137^Cs shows a small increase; (iii) the too low activity of ^137^Cs in surface soil in this area (Sabatier et al., 2008).

The three sediment cores were composed of successive layers of sand and organic matter that were more or less oxygenated. Three major sand layers (Sand 1–3 in Figure 3) were useful to correlate the cores. These sandy horizons might correspond to extreme meteorological events (wind storms, flood). Despite very good weather data in the area (Quillé, 2000), there are too many events that could correspond to these layers to be informative. However, the presence of *Af* cysts around 56 cm (introduced in 1970 in Aigues‐Mortes) shows coherence with the ^137^Cs peak at 62 cm, which is associated with the maximum radiation fallout of 1963–1964. These data confirmed that the gate closure was prior to this period. We were not able to detect the Chernobyl radiation peak of 1986 so we could not confirm whether the surface sediments were post‐1980s or not. Finally, cyst genotyping showed that most of the cores were post‐1970 (date of *Af* introduction in the saltern).

### 2.3 Using *Artemia* cysts from sediment cores

Our analysis of sediment cores in Aigues‐Mortes saltern and their cyst content revealed important conclusions for the use of *Artemia* in RE studies. First, it is possible to easily collect and purify cysts from sediments, in relatively large quantities. Second, in salterns, where many large *Artemia* populations are found worldwide, stratigraphic sampling is possible to provide a good knowledge of the sites. Third, core synchronization is possible, especially using the pattern of thick sandy horizons probably corresponding to extreme events (storms, floods). Fourth, despite a very high variability of the sediment grain size, dating is possible, especially combining an age control from ^137^Cs and the date of *Af* introduction in the saltern. Fifth, short DNA sequences can be readily obtained from cysts, even after several decades, which could be directly deep‐sequenced.

There are, however, also important shortcomings. First, hatchability of sedimented cysts is extremely low in sediments a few centimeters below surface [although we obtained some hatching in a similar core at the same site in a previous trial at 40 cm depth, unpublished results], which is a severe limitation. More intensive hatching tests may be required to determine if a small fraction of viable cysts can nevertheless be obtained for older samples, using tailored hatching protocols and perhaps using trials operated immediately after core extraction. Studying patterns of DNA repair in rehydrated cysts would also be particularly insightful to study this more precisely, as in Hespeels et al. (2014). Second, precise sediment dating may be difficult to obtain, due to the lack of a regular sedimentation pattern, as illustrated in our case study.

## 3 POSSIBLE APPLICATIONS OF *ARTEMIA* AS RE MODEL

**Figure 4:**
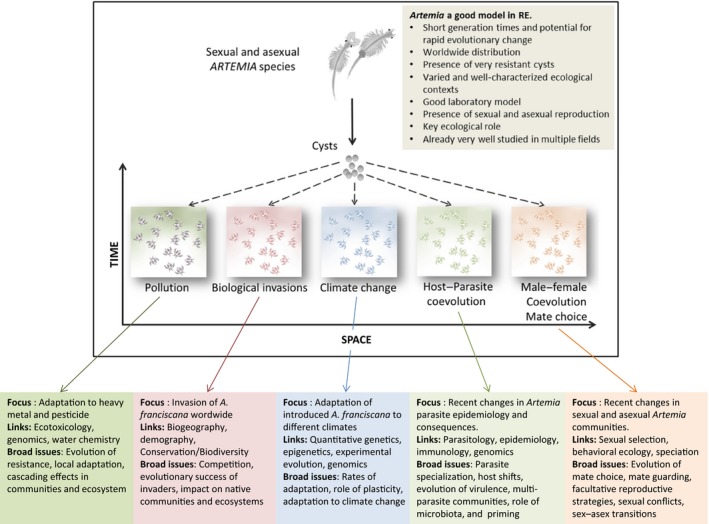
*Artemia*: a model system for resurrection ecology. Synopsis of key biological features and the main topics where the use of *Artemia* as a model system can contribute to important breakthroughs and can bridge gaps across fields

Only a few animal species have been used as models to conduct RE studies (Derry, Arnott, & Boag, 2010; Franks et al., 2008; Hairston et al., 1999; Roy Chowdhury, 2016; Weider et al., 1997). Finding new models from different ecosystems is of key importance to make progress to fully exploit this research strategy. We discuss here how *Artemia* can be used for RE studies (see overview in Figure 4). First, hypersaline environments where *Artemia* occur are essential parts of the biosphere (Mohebbi, 2010). They are common in dry regions (one*‐*third of the earth's land surface) and are distributed worldwide. These extreme environments are diverse (chemical composition, temperature regime) and replicated, allowing for large‐scale studies and comparison of different environmental stresses at different spatial and temporal scales. These systems have a simplified food web and low diversity (compared with, e.g., much more complicated freshwater ecosystems), making them ideal to study ecological and evolutionary dynamics. They are often human‐managed, which implies that there are detailed long‐term records of abiotic (salinity, temperature, oxygen, etc.) and biotic factors (birds, invertebrates, and phytoplankton communities, among others) that can be used to compare environmental change with evolutionary and community changes. They are often exposed to anthropogenic disturbance (Amat et al., 2007; Gajardo & Beardmore, 2012; Muñoz, 2010), therefore providing “natural experiments” that are crucial to understand the dynamics of adaptation to changing environments *in natura*, and on a long timescale. Hence, the study of *Artemia* populations offers many opportunities to study ecological and (co)evolutionary change. In the following, we discuss five topics, with specific field examples involving *Artemia* that could be particularly fruitful, especially with a RE strategy.

### 3.1 Biological invasions

One of the main components of anthropogenic global change is the introduction of invasive species (Vitousek, Antonio, Loope, & Westbrooks, 1996). Invasion and its multiple steps (Beard & Kulmatiski, 2012) are best studied with a historical perspective. As exemplified in the case of Aigues‐Mortes saltern mentioned above, the RE approach can be used to study these dynamics in detail. Hatching cysts from the past is particularly helpful to document postinvasion evolutionary changes in the invader and the invaded community. Because of their use in aquaculture (Sorgeloos, Dhert, & Candreva, 2001), cysts of the North American *Af* species have been introduced in many hypersaline systems, rapidly replacing native *Artemia* species worldwide (Amat et al., 2005, 2007). *Af* was introduced on to Pacific Islands and Brazil in the 1970s (Van Stappen, 2002) as well as in Europe (see above). It rapidly invaded the entire Mediterranean region (Amat et al., 2005, 2007; Green et al., 2005; Naceur, Jenhani, & Romdhane, 2010) and is now present in the Middle and Far East (Amat et al., 2007) and Australia (Ruebhart, Cock, & Shaw, 2008). This global invasion offers unique opportunities to study microevolutionary changes in *Af* (the invader) and various invaded communities (of *Artemia* and their associated parasites), a notoriously difficult issue (Mura et al., 2006; Suarez & Tsutsui, 2008). A RE approach would be particularly useful to measure fitness changes of the invader in different environments, as well as associated phenotypic and genomic changes. This information is not only relevant for fundamental reasons, but also because the understanding of the evolutionary processes that are involved in successful invasions can help to develop management strategies for their prevention or control (Sakai et al., 2001; Suarez & Tsutsui, 2008).

### 3.2 Global climate change

*Artemia* have been intentionally introduced in several parts of the world, for various economic reasons. Very often, *Artemia* were introduced to completely different biotic and abiotic conditions compared to their native habitats (e.g., in terms of temperature, water chemistry, predators, and parasites). Because these factors play a central role in the biology of *Artemia* (Van Stappen, 2002), they offer natural experiments to study adaptation in the field, especially when cyst time series are available. For example, *Af* from San Francisco Bay (California, USA) were introduced in new salterns in tropical areas (e.g., in Vietnam in 1982, Quynh & Lam, 1987). The climatic shift in these situations is close to +10°C for annual average temperature (Clegg, Jackson, Van Hoa, & Sorgeloos, 2000; Kappas, Abatzopoulos, Van Hoa, Sorgeloos, & Beardmore, 2004; Sebesvari, Le, & Renaud, 2011), which exceeds the most pessimistic climate change forecast for the 21st century (RCP8.5 Model predicts +6°C, IPCC 2013). These introductions can therefore be used to determine the extent to which species can tolerate and adapt to abrupt increases in temperature on a long‐term basis (hundreds of generations) and in the field. Temperature is a well‐known factor affecting the fitness of *Artemia* (Amat et al., 2007; Barata, Hontoria, Amat, & Browne, 1996; Browne & Wanigasekera, 2000). Clegg et al. (2000) showed that *Af* from Vietnam had greater thermal tolerance compared with individuals from San Francisco Bay and that these differences were transmitted to the following generations. These observations could be scaled up to measure rates of adaptation in a time series, as well as associated phenotypic and genotypic changes.

### 3.3 Global environmental pollution

Most studies in ecotoxicology focus on determining the effect of contaminants on different species, as well as on finding reliable biomarkers for environmental risk assessment. *Artemia* is extensively used in basic and applied aquatic toxicology (Persoone & Wells, 1987 for review), with a set of robust toxicity protocols (Artemia reference Center, ARC). Studying the evolutionary impact of man‐made change (various pollution, pesticides, heavy metal, etc.) has long been used to provide among the best detailed examples of adaptation (Bijlsma & Loeschcke, 2005; Bishop & Cook, 1981). However, a major limitation to these studies is that they require long‐term monitoring. Further, past or original populations are rarely available and adaptation is studied by comparing populations adapting to different habitats (“local adaptation,” Lenormand, 2002; Räsänen, Laurila, & Merilä, 2003). RE can be an extremely powerful strategy in this context. Several *Artemia* populations would be particularly interesting in that regard.

*Artemia* occur in two of the most metal‐polluted ecosystems in Europe, the Ria de Aveiro in Portugal, and the Odiel estuary in South Spain. Ria de Aveiro received a highly contaminated effluent discharged by a mercury cell chlor‐alkali plant from the 1950s until 1994 (Pereira et al., 2009). As a consequence, a total of 33 tons of mercury have accumulated in the wetland, with a significant amount of mercury being stored in sediments (Pereira, Duarte, Millward, Abreu, & Vale, 1998), and released in the saline water of the Ria. In the last decades, mercury discharge has been reduced and the quality of the water improved (Pereira et al., 2009) to reach levels that are today compatible with the EU environmental Directive 82/176/EEC 1982. This historical situation offers an interesting example where a RE approach could be developed to understand adaptation to realistic metal concentrations, as well as relaxation of selection (see also Piscia et al., 2012; for a similar approach). At the physiological and genetic levels, heavy metal‐induced detoxification mechanisms can be studied (Seebaugh & Wallace, 2004), such as those involving metal‐binding proteins (metallothioneins; it is a main metal detoxification strategy in *Artemia* and other aquatic organisms).

Another kind of pollution, widely occurring in *Artemia* habitats, is eutrophication. The Great Salt Lake (GSL) ecosystem provides an interesting situation to study biological responses to nutrient input with a RE approach. The lake receives high levels of industrial, urban, mining, and agricultural discharge. The construction of a railroad causeway in 1959 divided the lake into two water bodies, affecting the biogeochemistry and distribution of nutrients (Naftz et al., 2008). Reconstruction of changes in the sediment and water quality of GSL from the early 1700 to 1998 showed that the period from 1979 to 1998 was the most contaminated (Naftz et al., 2008). Comparative studies between the two parts of the lake and as well as during and before the most toxic period can increase our understanding of the evolutionary response of *Artemia* to nutrient pollution.

### 3.4 Host–parasite coevolution

Understanding host–parasite coevolutionary dynamics in the field is an important challenge. As we discussed above, many studies have used spatial data to infer patterns of host–parasite interactions, focusing on local adaptation (Greischar & Koskella, 2007). Studying host–parasite coevolution requires long‐term data sets, which is not always feasible. *Artemia* is the (intermediate) host of a very well‐characterized community of parasites and pathogens, including helminths with complex life cycles through avian final hosts (Georgiev et al., 2005; Vasileva et al., 2009), horizontally transmitted microsporidians (Martinez, Vivares, & Bouix, 1993; Martinez et al., 1992; Rode, Landes, et al., 2013; Rode, Lievens, et al., 2013; Rode, Lievens, Flaven, et al., 2013), yeasts (Codreanu & Codreanu‐Balcescu, 1981), viruses, and bacteria (Crab, Lambert, Defoirdt, Bossier, & Verstraete, 2010; Li, Zhang, Chen, & Yang, 2003; Soto‐Rodriguez, Roque, Lizarraga‐Partida, Guerra‐Flores, & Gomez‐Gil, 2003). Some of these parasites can produce resting stages (e.g., vibrio species which are found in sediments, Williams & LaRock, 1985) which may eventually be recovered from the sediments together with *Artemia* cysts. Many of these parasites strongly affect host fitness through castration, physiological and behavioral manipulation and increased predation (Amat, Gozalbo, Navarro, Hontoria, & Varó, 1991; Redón, Amat, Sánchez, & Green, 2015; Rode, Lievens, Flaven, et al., 2013; Sánchez, Hortas, Figuerola, & Green, 2009; Sánchez et al., 2012; Sánchez, Thomas, et al., 2009) and are expected to impose strong selective forces potentially leading to rapid evolution of defense mechanisms. So host and parasite populations from different times can be resurrected and used in time‐shift and cross‐infection experiments to provide important insight on long‐term patterns of coevolution in the field (Decaestecker et al., 2007). Furthermore, *Artemia* immunity (as measured, e.g., by phenoloxidase activity) could be tracked through time in response to the abundance of parasites. The latter usually correlates well to the density of final hosts (Sánchez et al., 2013), which is well documented. For instance, the Mediterranean population of flamingos (*Phoenicopterus roseus*) has exponentially increased over the last 30 years (Rendón, Green, Aquilera, & Almaraz 2008), and it is expected to be associated with a concomitant increase in the prevalence of the cestode *Flamingolepis liguloides* in *Artemia* (the most common avian cestode using *Artemia* as intermediate host and flamingos as final hosts). The introduction of *Af* in new regions, which is generally associated with the loss of parasites (Georgiev, Sánchez, Vasileva, Nikolov, & Green, 2007; Sánchez et al., 2012), could provide the opposite scenario with a “relaxed selection” for resistance.

### 3.5 Male–female coevolution and mate choice

The availability of cysts from different time periods can be used to follow other patterns of coevolution, such as between males and females. The long‐term consequence of sexual conflicts can be studied experimentally in the laboratory on model organisms such as *Drosophila* (Arnqvist & Rowe, 2005). They are, however, very difficult to study in the field. RE in bisexual *Artemia* species can be used for this purpose. For instance, Rode et al. (2011) studied male–female coevolution in *Af*, using a ~160‐generation (c.a. 23 years) time series. They found that females had better survival and longer interbrood intervals when mated with their contemporary males compared to when mated with males from their future or their past, demonstrating fast male–female coevolution *in natura*. Other evolutionary patterns of mating/reproductive traits could be fruitfully studied using RE in *Artemia*. In particular, female facultative sex‐ratio adjustment or male mate discrimination could be studied in parthenogenetic native populations recently invaded by *Af* (Lievens, Henriques, Michalakis, & Lenormand, 2016).

## 4 CONCLUSION

*Artemia* offers multiple avenues for RE research. New “omics” approaches (genomics, transcriptomics, proteomics, or metabolomics) and the development of *Artemia* as model system in genomics (De Vos, 2014) would add a new dimension to the use of *Artemia* in RE, notably to exploit the DNA record that can be obtained in sedimented cysts. Coupled with experimental evolution studies, it would provide resurrection ecologists with stronger insights into a large suite of fundamental questions (local adaptation, host–parasite coevolution, reproductive system evolution), as well as firmer predictions about the effects of global change on organisms, communities, and ecosystems.

## DATA ARCHIVING STATEMENT

Data available from the Dryad Digital Repository: https://doi.org/10.5061/dryad.td0g3.
