# Supplementary material for: Resurrection ecology in Artemia
Source: Evol Appl. 2017 Oct 23;11(1):76–87. doi: 10.1111/eva.12522 (PMC5748519; doi:10.1111/eva.12522)

Suplementary material

Resurrection Ecology in *Artemia*

Thomas Lenormand^1^, Odrade Nougué^1^, Roula Jabbour-Zahab^1^, Fabien Arnaud^2^, Laurent Dezileau^3^, Luis-Miguel Chevin^1^ and Marta I. Sánchez^4^

^1^ CEFE UMR 5175, CNRS - Université de Montpellier - Université Paul-Valéry Montpellier - EPHE -1919 route de Mende, F-34293 Montpellier, CEDEX 5, France

^2^ Laboratoire EDYTEM - UMR 5204 du CNRS - « Environnements, Dynamiques et Territoires de la Montagne ». Campus scientifique, Université de Savoie F-73376 Le Bourget du Lac Cedex

^3^ Géosciences Montpellier, UMR 5243, Université de Montpellier, Place Eugène Bataillon
34095 Montpellier cedex 05, France

^4^ Estación Biológica de Doñana (CSIC), Avda. Américo Vespucio s/n 41092, Sevilla, Spain

## Core sampling and cyst typing

For the first core, a hole of 1 m deep was dug with one vertical side. To obtain large quantity of cysts, 19 cuboids of sediments (each 16 x 16 x 3.5 cm) were sampled from the vertical side of the hole, divided into two equal parts and placed in individual hermetic bags. Cysts in the core were dehydrated and then floated on brine (120g/L) to separate them from the sediments. Hatching tests were performed using standard protocols after decapsulation (Rode et al., 2011) in layers n°1, 2, 5, 9, 15, 17, 19 and surface cyst samples. For each core horizon, the DNA from 20 individual cysts was extracted using 15µL of E buffer (HotSHOT, Sigma-Aldrich, ref. E7526), then placed in a thermocycler (95°C for 10 min plus 20°C for 10 min). Finally, the extract was diluted with 25µL of sterile water. Two species-specific microsatellite markers were used to type each individual cyst (Ap02 and Af03 for *Ap* and *Af*, Muñoz et al., 2008b). Briefly, the PCR mix was made of 5 µL of Multiplex buffer, 1 µL of both forward and reverse primers [2 µM], 2 µL of sterile water and 1 µL of DNA sample. Thermocycler program was initial denaturation (15 min, 95°C), 30 cycles (denaturation 30sec, 94°C; annealing 90sec, 60°C;extension 60sec, 60°C), and final extension (30sec, 60°C). PCR products were separated on a 2% agarose gel for 30 min for genotype identification.

## Core sampling and synchronization

Manual corer consisting in 1m long PVC tubes (9cm diameter) were pushed directly through the sediments. A pipe connected to an air compressor was attached at the bottom of the tubes. Air injection at the bottom of the core hence facilitated the extraction of the tube (the viscosity of the wet sediment prevents extraction only using vacuum force). Cores were opened, photographed, and their chemical composition characterized using an Avaatech X-ray Fluorescence (XRF) CoreScanner, which performs non-destructive analysis of elements from magnesium (Mg) right through to uranium (U) along the core axis. It allows synchronizing the different cores, searching for sedimentation cycling (year markers), and identifying special sedimentation events. We performed a Principal Component Analysis (PCA) to analyze the variation of diverse elements that are known to be associated with different types of sediments (Supplem. Fig S4). Among measured elements sulfur and copper are more often associated with anoxic environment whereas Fe and Mn generally vary with the amount of their oxide form, i.e., thus tracking periods of better oxygenation. Potassium (K) and silicon (Si) on their own track the input of terrigenous material which can be clayey or sandy, respectively. Here we focused on the use of another element, bromine (Br) which is classically associated to organic matter in lake and marine sediment. In our cores sandy layers are clearly associated to drops in Br content confirming it tracks the balance between authigenic organic matter and terrigenous mineral input. Comparing Br curves hence permits the synchronization of cores (Fig. 2, Supplem. Fig. S5). Synchronizing cores is essential for analyses (e.g. molecular biology, Gamma spectrometry) that involve destructive protocols.

## Sediment dating

Dating of sedimentary layers was carried out using lead (^210^Pb) and cesium (^137^Cs) isotopes methods on a centennial timescale. Both nuclides together with uranium (U), thorium (Th), and radium (^226^Ra) were determined by gamma spectrometry at the Géosciences Montpellier Laboratory (University of Montpellier). The 1-cm-thick sediment layers were finely crushed after drying, and transferred into small gas-tight PETP (polyethylene terephtalate) tubes (internal height and diameter of 38 and 14 mm, respectively), and stored for more than 3 weeks to ensure equilibrium between ^226^Ra and ^222^Rn. The activities of the nuclides of interest were determined using a Canberra Ge well detector and compared with the known activities of an in-house standard. Activities of ^210^Pb were determined by integrating the area of the 46.5-keV photo-peak. ^226^Ra activities were determined from the average of values derived from the 186.2-keV peak of ^226^Ra and the peaks of its progeny in secular equilibrium with ^214^Pb (295 and 352 keV) and ^214^Bi (609 keV). In each sample, the ^210^Pb excess activities (^210^Pb_ex_) were calculated by subtracting the (^226^Ra supported) activity from the total (^210^Pb) activity (Dezileau et al., 2016).

^210^Pb (T1/2 = 22.3 yr) is a widely used radiotracer in the study of sedimentary environments on a temporal scale of 100–150 years (e.g., Robbins and Edgington, 1975). This naturally occurring radionuclide is present in sediments after formation by decay of ^222^Rn (T1/2 = 3.8 d) in the atmosphere and the water column (^210^Pb excess) and in situ decay of ^226^Ra (T1/2 = 1600 yr). Age models based on ^210^Pb activity profiles in sediment cores can be confirmed by independent time-stratigraphic markers, such as artificial radionuclides (e.g., ^137^Cs, ^241^Am), which allows us to discriminate the influence of postdepositional processes such as mixing. The onset of ^137^Cs presence in sediments corresponds to the beginning of the atmospheric nuclear tests in the 1950s, and the highest concentrations correspond to maximum atmospheric activities due to nuclear tests (1963, see Ayrault et al., 2009; Sabatier et al., 2010 for details).

Laser grain-size analyses were done with a Beckmann-Coulter LS13320 Particle Size Analyser (Géosciences Montpellier Laboratory). Grain-size analyses were performed on the sequence with an average interval of 1 cm. Each sample was initially sieved at 1 cm, suspended in deionised water and gently shaken to achieve disaggregation. Ultrasound was used to avoid particle flocculation of sediment in the fluid module of the granulometer. For each sample, a small homogeneous amount of sediment was mixed in deionized water then sieved at 1.5 mm diameter before pouring in the Fluid Module of the Particle Sizer to obtain an optimal obscuration rate between 7 and 12% in the Fraunhofer optical cell. The time of background and sample measurement was set to 90 s and sonication was applied during the measurement of the sample in order to improve the dispersion of fine particles in the fluid. Each sample was measured twice and the good repeatability of measurement was verified according to the statistics from the international standard ISO 13320-1.

Ayrault, S., Lefèvre, I., Bonté, P., Priadi, C., Carbonaro-Lestel, L., Mouchel, J., Lorgeoux, C., Djouarev, I., Gasperi, J., Moilleron, R. & Tassin, B. (2010). *Archives sédimentaires, témoignages de l'histoire du développement du bassin*, 65.

**Dezileau L**., Lehu R., Lallemand S., Hsu S-K., Babonneau N., Ratzov G., Lin A.T., Dominguez S., (2016) Historical reconstruction of submarine eartquakes using 210Pb, 137Cs and 241Am turbidite chronology and radiocarbon reservoir age estimation off East Taiwan. Radiocarbon, *58*, 25-36.

Muñoz, J., Green, A.J., Figuerola, J., Amat, F. & Rico, C. (2008b). Characterization of polymorphic microsatellite markers in the brine shrimp *Artemia* (Branchiopoda: Anostraca). *Molecular Ecology Resources* 9, 547–550.

Robbins, J. & Edgington, D. (1975). Determination of recent sedimentation rates in Lake Michigan using Pb-210 and Cs-137. *Geochimica Cosmochimica Acta,* 39, 285–304.

**Sabatier, P.,** Dezileau, L., Blanchemanche, P., Siani, G., Condomines, M., Bentaleb, I. & Piquès, G. (2010). Holocene variations of radiocarbon reservoir ages in a Mediterranean lagoonal system. *Radiocarbon,* 52, 91-102.

# Supplementary figures

**Figure S1**. *Artemia* cysts (in brown colour) accumulated along the shore of a pond in Odiel saltpans (SW Spain)


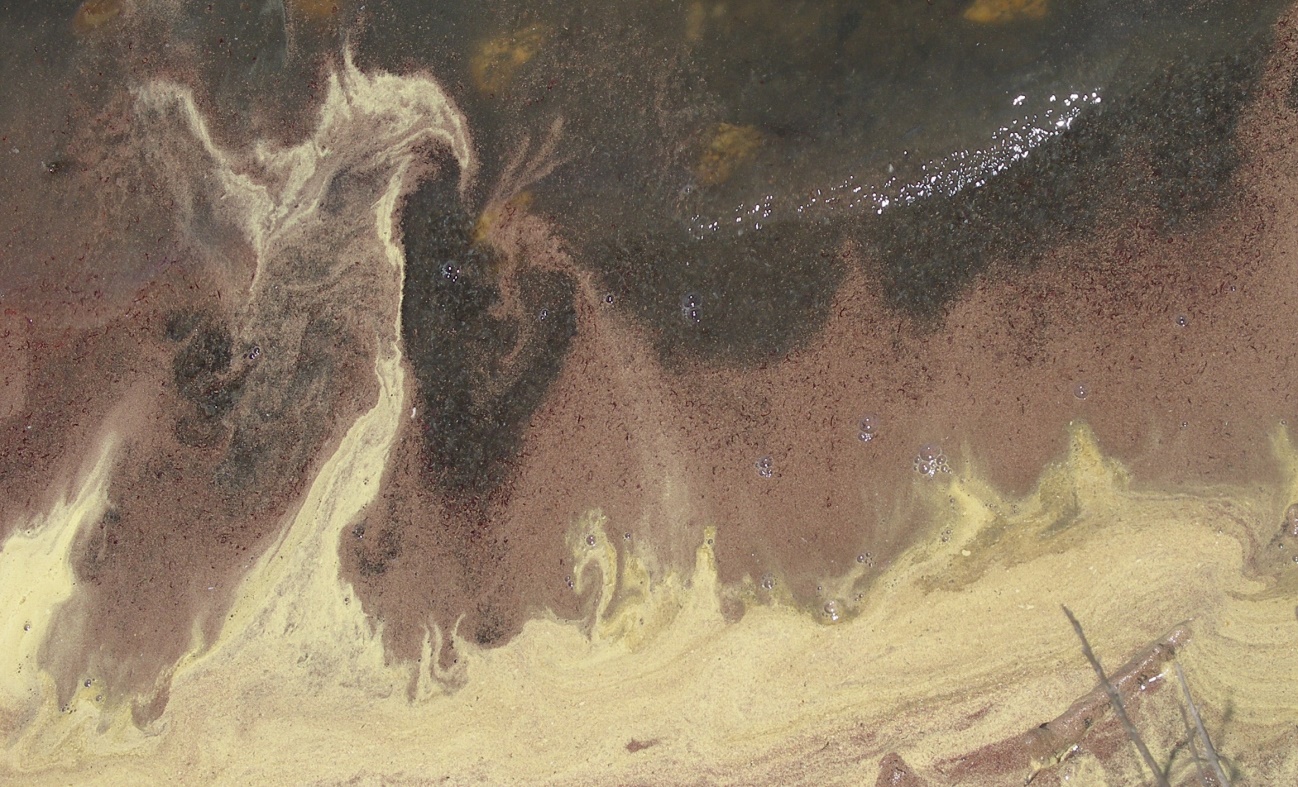


**Figure S2: Sampling site.** Ancient sluice gate (blue lines) close to l’Abbé (43°31'24.72"N, 4°14'12.67"E) in Aigues-Mortes saltern. The large orange square represents ABB12 core, while the three smaller squares represent the cores ABB13_P5, P6, P7 (from left to right).


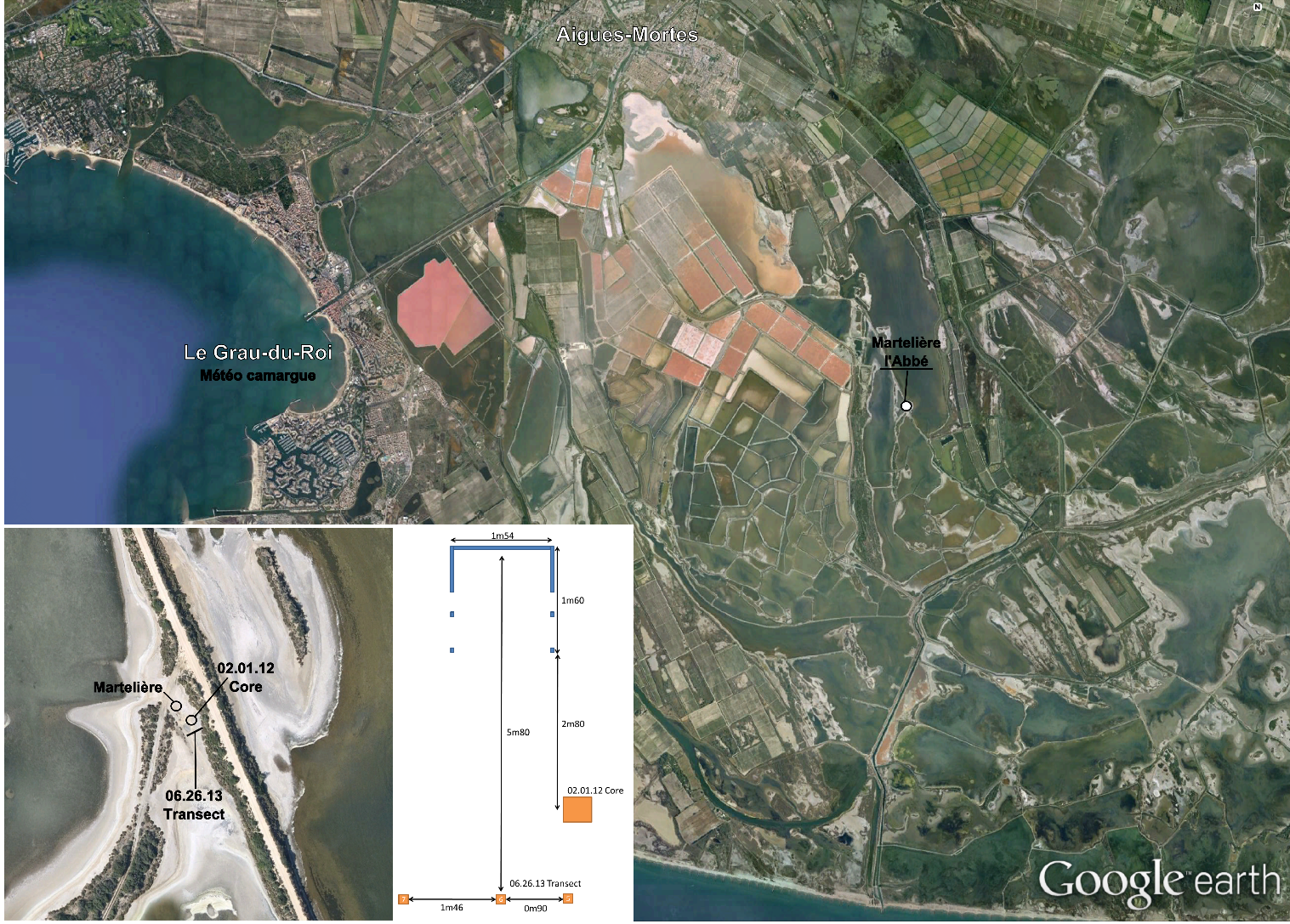


**Figure S3 : Size of DNA fragment extracted from cysts at different sediment depths (**9cm, 45cm, 66cm). The black arrow on the ladder indicates 100 bp.


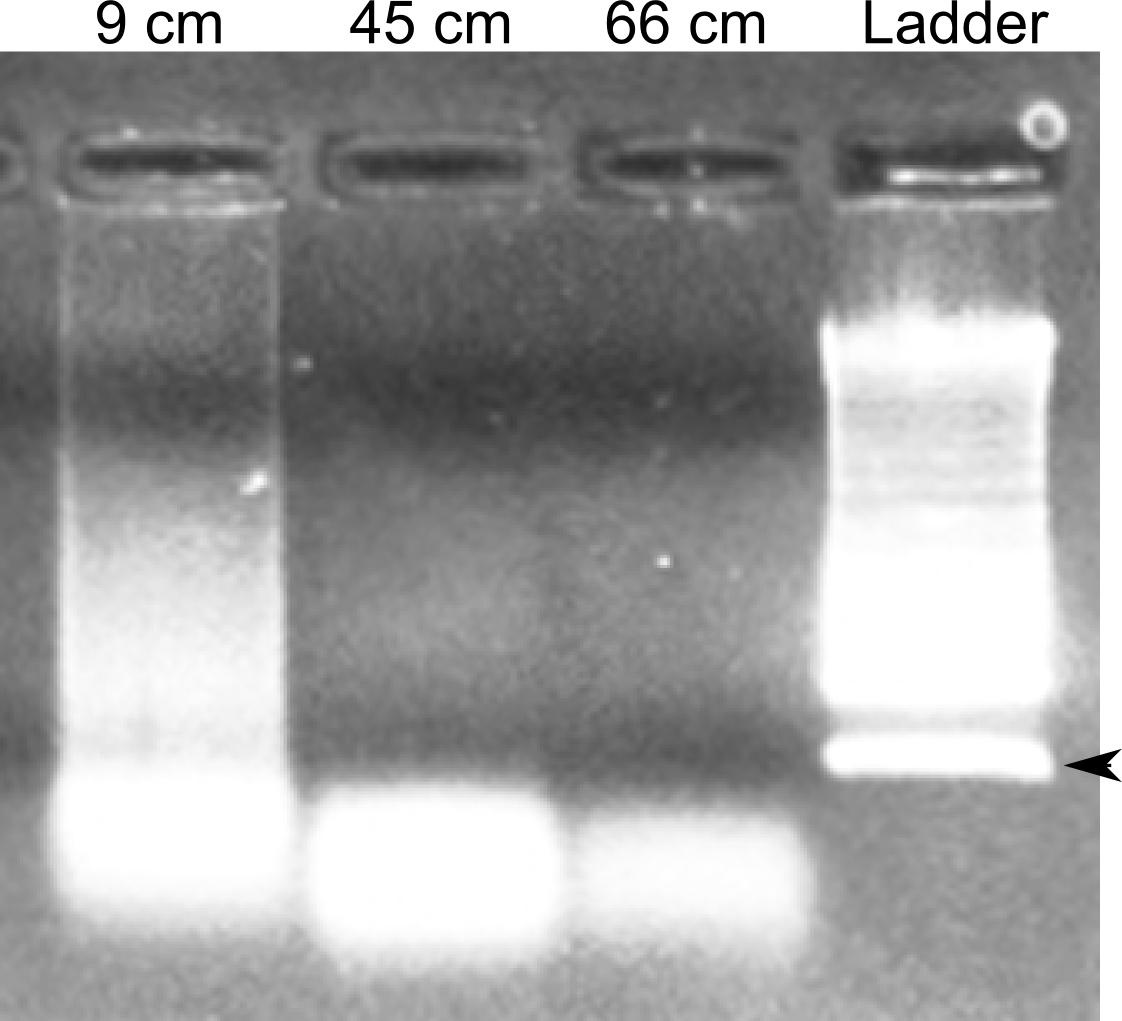


**Figure S4: PCA of XRF CoreScanner results for ABB13_P5.** Chemical element associated with clay (triangle; Rb: rubidium), sandstone (square, K: Potassium; Si: Silicon) and organic matter of high (open circle; Fe: iron; Ca: calcium; Ti: titanium; Mn: manganese) or low oxygenation (full circle; S: sulfur; Br: bromine; Cu: copper; Sr: strontium).

Br

Ti

Mn

Rb

K

Fe

Ca

Cu

S

Sr

Si

**Figure S5: Visual correlation of sediment cores.** The dotted/shaded zone corresponds to the bottom of cores ABB13_P6 and ABB13_P7 where sediments were stirred. The yellow shaded zones correspond to clear sandy areas.


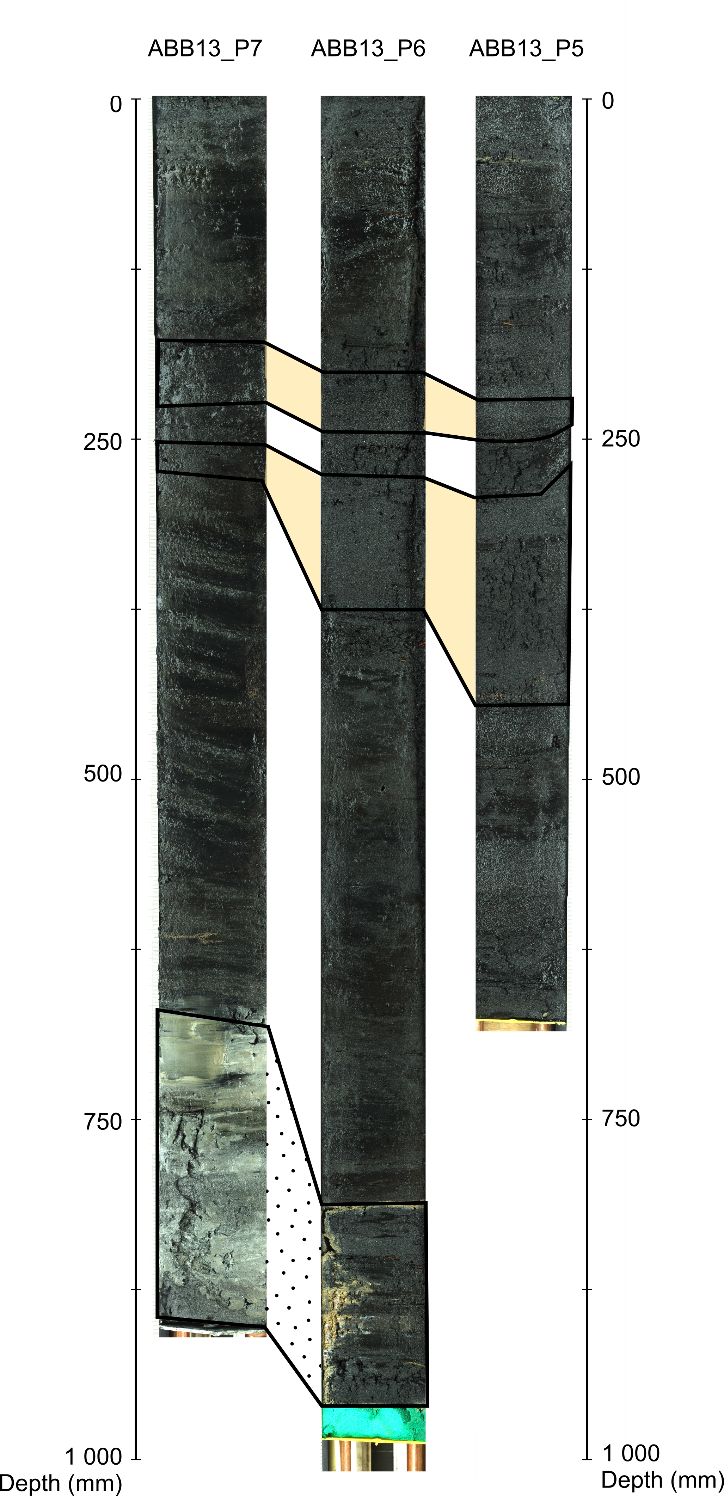

Supplement: Supplementary file 1 [file EVA-11-76-s001.docx]
